# Reference Values for Skeletal Muscle Mass – Current Concepts and Methodological Considerations

**DOI:** 10.3390/nu12030755

**Published:** 2020-03-12

**Authors:** Carina O. Walowski, Wiebke Braun, Michael J. Maisch, Björn Jensen, Sven Peine, Kristina Norman, Manfred J. Müller, Anja Bosy-Westphal

**Affiliations:** 1 Institute for Human Nutrition and Food Science, Christian-Albrechts-University Kiel, 24105 Kiel, Germany; cwalowski@nutrition.uni-kiel.de (C.O.W.); wbraun@nutrition.uni-kiel.de (W.B.); mmueller@nutrfoodsc.uni-kiel.de (M.J.M.); 2 seca gmbh & co. kg., Hammer Steindamm 3-25, 22089 Hamburg, Germany; michael.maisch@seca.com (M.J.M.); bjoern.jensen@seca.com (B.J.); 3 Institute for Transfusion Medicine, University Hospital Hamburg-Eppendorf, 20246 Hamburg, Germany; s.peine@uke.de; 4 Department of Nutrition and Gerontology, German Institute of Human Nutrition, Potsdam-Rehbruecke, 14558 Berlin, Germany; kristina.norman@dife.de; 5 Charité-Universitätsmedizin Berlin, Corporate Member of Freie Universität Berlin, Humboldt-Universität zu Berlin and Berlin Institute of Health, 13347 Berlin, Germany

**Keywords:** sarcopenia, sarcopenic obesity, skeletal muscle mass, skeletal muscle area, skeletal muscle mass index, appendicular skeletal muscle mass index, fat-free mass index

## Abstract

Assessment of a low skeletal muscle mass (SM) is important for diagnosis of ageing and disease-associated sarcopenia and is hindered by heterogeneous methods and terminologies that lead to differences in diagnostic criteria among studies and even among consensus definitions. The aim of this review was to analyze and summarize previously published cut-offs for SM applied in clinical and research settings and to facilitate comparison of results between studies. Multiple published reference values for discrepant parameters of SM were identified from 64 studies and the underlying methodological assumptions and limitations are compared including different concepts for normalization of SM for body size and fat mass (FM). Single computed tomography or magnetic resonance imaging images and appendicular lean soft tissue by dual X-ray absorptiometry (DXA) or bioelectrical impedance analysis (BIA) are taken as a valid substitute of total SM because they show a high correlation with results from whole body imaging in cross-sectional and longitudinal analyses. However, the random error of these methods limits the applicability of these substitutes in the assessment of individual cases and together with the systematic error limits the accurate detection of changes in SM. Adverse effects of obesity on muscle quality and function may lead to an underestimation of sarcopenia in obesity and may justify normalization of SM for FM. In conclusion, results for SM can only be compared with reference values using the same method, BIA- or DXA-device and an appropriate reference population. Limitations of proxies for total SM as well as normalization of SM for FM are important content-related issues that need to be considered in longitudinal studies, populations with obesity or older subjects.

## 1. Introduction

Beyond the well-established role of ageing associated loss in skeletal muscle mass (SM) (primary sarcopenia) as a risk factor of frailty, morbidity and mortality in older people, a low SM is observed as a result of diseases like malignant cancer, chronic obstructive pulmonary disease, heart failure and renal failure (secondary sarcopenia [1]) and is also an emerging prognostic marker in a number of diseases [2,3,4,5,6,7,8,9,10,11,12]. The etiology for sarcopenia as a risk factor might be partly explained by the correlation between SM and cardiac, respiratory or immune function but remains to be investigated further in order to understand the preventative and therapeutic potential of SM. Muscle not only functions as the major tissue for insulin-stimulated glucose uptake, amino acid storage and thermoregulation, but is also secreting a large number of myokines that regulate metabolism in muscle itself as well as in other tissues and organs including adipose tissue, the liver and the brain [13,14]. The recent popularity of SM outpaced the interest in fat mass (FM) that only has a limited and inconsistent impact on morbidity and mortality [15,16]. The assessment of SM by segmentation of continuous whole body magnetic resonance imaging (MRI) is considered as the gold standard [17]. However, this method is too cumbersome and expensive for clinical practice and is even rarely used in studies with larger sample sizes [17,18]. Instead, single slices at different reference sites measured by MRI or obtained from routine computed tomography (CT) examinations are taken as a proxy for the total tissue volume (e.g., L3 muscle cross-sectional area [17,19]). Most commonly, dual X-ray absorptiometry (DXA) is used to assess appendicular lean soft tissue (ASM, the sum of lean soft tissue from both arms and legs) or fat-free mass (FFM, total lean soft tissue plus bone mineral mass or body weight minus FM) as a proxy for SM. More simple and even non-invasive, the output of bioelectrical impedance analysis (BIA) depends on the reference method used to generate the BIA algorithm and can be FFM [20], ASM, e.g., [21,22,23] or even SM, e.g., [24,25,26,27]. 

To facilitate comparison between studies and to evaluate individual results for SM in patients, it is important to understand the differences between parameters and cut-offs for SM. These differences are not only method inherent but also depend on characteristics of the study population (e.g., ethnicity, age and disease). Device-specific characteristics by different manufacturers determine the validity and precision of parameters for SM. In addition, the available reference values differ with respect to parametric normalization (linear regression or indexing) to account for body size. Further complexity to the definition of a normal SM is derived from the concept of sarcopenic obesity [28]. Since high levels of FM may adversely affect the quality and function of SM [29,30], a normal SM may also depend on the amount of FM. 

Different professional associations have published definitions of sarcopenia based on an estimate of SM and impaired muscle strength and/or physical performance [31,32,33,34,35,36,37], but no consensus definition has yet been reached. The aim of this review is not to provide an optimal diagnosis of sarcopenia but to compare current definitions of a low SM considering the impact of the underlying methodological assumptions, limitations and normalization of SM parameters for height, weight, body mass index (BMI) or FM.

## 2. Methods

In order to identify reference values for SM, seven consensus reports were reviewed [31,32,33,34,35,36,37]. Further studies were identified through reference lists and a search for relevant articles based on the keywords “sarcopenia”, “low muscle mass”, “cut-off sarcopenia”, “reference value sarcopenia”, “sarcopenic obesity”. Only parameters of SM normalized for height, weight, BMI or FM were considered. To be included in this article, studies were required to contain the following information: method of SM assessment (device), cut-off points for SM and description of the reference population including geographical location, sample size, distribution between sexes and age (range and/or standard deviation (SD) ± mean). Only English language articles were considered. Therefore, 64 studies were identified that met the inclusion criteria. Main reasons for the exclusion of articles were duplicate analyses conducted on the same reference population (only the first published paper was included), a missing normalization of reference values, a sample size <200 subjects (sample size <200 subjects will not be representative for both sexes, all ages and BMI-groups), the use of anthropometric measures to determine a low SM and the adoption of previously published cut-offs regarding SM and obesity.

### Study Characteristics

Studies that met the inclusion criteria were published between 1998 and 2019 and were performed in 21 countries. The sample size of the individual studies ranged from 200 to 38,099 subjects with an age range between 18 and >90 years. In 36 studies, the authors clearly indicated that the reference population included healthy individuals. 

## 3. Results

Published cut-off points for a low SM normalized by height are presented in Table 1, Table 2 and Table 3 stratified by DXA, BIA and CT. In the majority of studies (14 of 32), SM was measured by DXA using lean soft tissue from the arms and legs normalized by height^2^ given as appendicular skeletal muscle mass index (ASMI) [22,38,39,40,41,42,43,44,45,46,47,48,49,50]. One study [40] used DXA-derived ASM to predict whole body SM measured by MRI using the equation by Kim et al. [51] that was validated in an ethnically diverse sample of healthy men and women. The range of published cut-off values for ASMI by DXA (without considering different classes of sarcopenia) was 5.86–7.40 kg/m^2^ in men and 4.42–5.67 kg/m^2^ in women. 

With ten studies, the second most commonly used method underlying published SM reference values was BIA [21,22,23,24,25,26,52,53,54,55]. To measure SM by BIA, five studies have used the BIA-equation by Janssen et al. [56] to predict SM [24,25,26,53,55]. This BIA-equation was developed and cross-validated against whole body MRI in a sample of 269 Caucasian men and women aged 18 to 86 years with a BMI of 16-48 kg/m^2^ using a model 101B BIA analyzer (RJL Systems, Detroit, MI, USA) [56]. The authors reported that the BIA-equation is applicable for Caucasian, African-American, and Hispanic populations but has not been validated for the estimation of SM in Asian populations. One study calculated SM by multiplying BIA-derived FFM with a constant factor (0.566) derived from comparison with SM estimates by 24 h creatinine excretion in healthy subjects [52]. The range of cut-offs for ASMI by BIA was 6.75–7.40 kg/m^2^ in men and 5.07–5.80 kg/m^2^ in women, whereas cut-offs for skeletal muscle mass index (SMI) by BIA validated against MRI ranged between 7.70 and 9.20 kg/m^2^ in men and 5.67 and 7.40 kg/m^2^ in women (without considering severity of sarcopenia). 

Nine studies used standard diagnostic CT to determine SM cut-off points for single slices [57,58,59,60,61,62,63,64,65]. Skeletal muscle area (SMA) at the level of the third lumbar vertebra (L3 SMA; L3 SMI = L3 SMA/height^2^, cm^2^/m^2^) was used in three studies on patients with cancer [62,64,65]. Cut-off points ranged between 36.00 and 43.20 cm^2^/m^2^ in men and 29.00 and 34.90 cm^2^/m^2^ in women. Six studies determined sex-specific cut-offs for SM by CT in healthy populations, thereof five in organ donors [57,58,59,60,61,63]. L3 SMI was used in four studies on healthy subjects [57,58,59,60] and three studies with a healthy reference group used CT imaging at the L3 level to measure the psoas muscle mass area (L3 PMA; L3 psoas muscle index (PMI) = L3 PMA/height^2^, cm^2^/m^2^) [57,61,63]. In healthy populations, cut-off values for L3 SMI ranged between 36.54 and 45.40 cm^2^/m^2^ in men and 30.21 and 36.05 cm^2^/m^2^ in women, whereas thresholds for L3 PMI were 2.63-6.36 cm^2^/m^2^ for men and 1.48–4.00 cm^2^/m^2^ for women.

### Combination of Measures for Muscle mass and Obesity

Table 4 shows reference values of 34 publications for a low SM in combination with different measures of obesity. Cut-offs for a low SM were mostly determined by DXA or BIA, whereas only a few studies reported CT-defined cut-offs in combination with obesity criteria. SM parameters were commonly normalized for height squared or given as % of body weight. In addition, two studies adjusted ASM for BMI [66,67]. Alternative parameters were FM/FFM ratio [68], visceral fat area/thigh muscle area ratio (VFA/TMA) [69] and fat mass index (FMI) in combination with fat-free mass index (FFMI) [70].

Prado et al. [71] published CT-derived SMI cut-offs determined in a population of obese (BMI ≥ 30 kg/m^2^) Canadians with tumors of the respiratory or gastrointestinal tract. In 2013, this CT database was extended by Martin et al. [72] and low SM reference values were reported for subjects with normal weight and overweight according to BMI classifications. In both studies, optimal stratification was used to determine the threshold of mortality. Many studies adopted the criteria proposed by Prado et al. [71] and Martin et al. [72] (e.g., [73,74,75]). Only one further study developed BMI-dependent reference values for SM [76]. Although some studies referenced the cut-offs by Prado et al. [71], reported thresholds differ from the original work (e.g., [77,78]). These reported values were then cited in further studies [79].

In most studies, obesity was defined as BMI ≥ 30 kg/m^2^ [71,76,80,81]. Alternative BMI thresholds were 27.5 kg/m^2^ [82,83], 27 kg/m^2^ [84], 25 kg/m^2^ [72,85,86,87,88,89,90] or 23 kg/m^2^ [91]. Furthermore, sex and ethnic-specific waist circumference (WC) thresholds for central obesity were considered [44,84,92,93,94,95]. Other criteria include %FM [50,81,96,97,98,99,100,101], visceral fat area [73] or fat-muscle ratios like visceral fat area (VFA) to total abdominal muscle area (TAMA) [74]. 

Table 5 displays cut-offs and average values for body composition stratified into groups of subjects with underweight, normal weight, overweight and obesity. Cut-offs for FMI_DXA_ were released by the National Health and Nutrition Examination Survey (NHANES; [102]) and respective BMI-dependent normal values for FFMI_DXA_ were calculated as BMI minus FMI. For each given BMI displayed in Table 5, corresponding normal value for SMI_MRI_ were calculated using a stepwise regression analysis (SMI_MRI_, men = 0.479 × FFMI_DXA_ −0.017 × age + 0.683 and SMI_MRI_, women = 0.348 × FFMI_DXA_ − 0.011 × age + 1.971) in a healthy Caucasian population. In addition, respective values for SMI_BIA_ validated against MRI were generated based on a young and healthy Caucasian population using linear regression analysis (SMI_BIA_, men = 0.168 × BMI + 5.49 (R^2^ = 0.53, standard error of estimate (SEE) = 0.514) and SMI_BIA_, women = 0.159 × BMI + 3.72 (R^2^ = 0.61, SEE = 0.465)). Adjacent to the average SMI_BIA_ (median) for each BMI, cut-offs with two SDs below the sex-specific mean of the young and healthy population were shown. 

## 4. Discussion

SM has evolved as the most promising body composition parameter associated with health risk in ageing and many chronic diseases [1]. Evaluation of SM is complicated by a variety of available methods that provide different outcome parameters as a proxy for total body SM. Therefore, it is important to have accurate reference values that apply to the patient or population under study as well as to the respective body composition method. In this review, we identified multiple published reference values for discrepant parameters of SM (Table 1, Table 2, Table 3 and Table 4), discussed the differences in the underlying assumptions and limitations as well as different concepts for normalization of SM parameters for height, weight, BMI or FM.

Imaging technologies are thought to provide the best assessment of SM. Briefly, segmentation of transversal images by special software (e.g., SliceOmatic Tomovision, version 4.3; Montreal, Québec, Canada) results in muscle areas that are multiplied by the correspondent slice thickness to calculate muscle volume [27] that is transformed to SM by assuming a constant density (1.04 kg/L) of adipose tissue-free SM [103]. Muscles at the head, hands and feet are commonly neglected in this approach. The precision of whole body SM_MRI_ is high (intra-observer coefficient of variation = 1.8% [104]). Reference data for total SM based on the gold standard whole body MRI (Table 5) are scarce due to high costs and cumbersome image-segmentation [17,18]. However, whole body MRI was integrated in the assessment of current large and representative national databases like the UK biobank [105] or the national cohort (NAKO) in Germany [106]. Future evaluation of these databases will provide the basis of statistically derived normal values whereas prospective investigation of mortality or correlation with frailty, fracture risk, glucose or amino acid metabolism would allow to establish even more meaningful disease-specific cut-offs. 

Instead of whole body imaging, reference values for L3 single slices are frequently published (Table 3 and Table 4), especially in patients where CT images are routinely applied for cancer staging. The use of these cut-offs may be specific for the population studied and transferability of the results to other patient groups needs to be investigated. Radiation exposure is a major limitation that confines the application of CT to individual transversal images or the secondary analysis of routine clinical measurements. As a further drawback, clinical CT protocols for L3 are not standardized across hospital sites. SMA at L1, L2, L4, L5, and the thoracic vertebra T12, T11, and T10 were reported to be suitable alternatives to SMA measured at L3 [58]. Nonetheless, there are also advantages of CT images with a high resolution and precision of the measurement. Most studies report the precision of single slice CT scan analysis to range between 1% and 2% [107]. Thus, automated segmentation is facilitated by using a characteristic range of Hounsfield units for fat-free muscle tissue [107,108]. CT can also differentiate individual muscle or muscle groups and can thus for example investigate the impact of pectoralis muscle area for survival at the Intensive Care Unit [12] because respiratory musculature may determine weaning from mechanical ventilation. On the other hand, characteristic changes in the Hounsfield distribution of muscle can reveal qualitative changes of the tissue (e.g., fatty infiltration or edema) that have been found to be of prognostic value [71].

DXA is the most commonly used method for assessment of SM (Table 1). Lean soft tissue at the arms and legs (ASM) is highly correlated with muscle volume derived from imaging studies (correlation coefficients ranging from 0.77 to 0.97 for both, whole body and regional scans [51,109,110,111,112,113,114,115]). However, only 44% of total lean soft tissue is derived from extremities (unpublished results) and only part of total lean soft tissue is SM. Therefore, SM measured by DXA is considerably higher when compared with muscle volume measured by imaging technologies [27,116]. Precision errors for total ASM are reported to be low (1–3%), device specific and depend on population characteristics like age or prevalence of obesity [117].

BIA can assess SM, ASM or FFM, depending on the reference method used to generate the BIA-algorithm. The choice of the BIA-algorithm not only depends on the desired target-parameter but also on the agreement between the BIA-device or reference population used to generate the BIA-algorithm and the BIA-device and patient characteristics to be evaluated [118]. However, in two studies, the equation by Janssen et al. [56] that is not suitable for Asians was used to predict SM in Asian populations [53,55] with only one study providing a validation in 41 Taiwanese people (age: 20–99 years; BMI: 17.6–34.6 kg/m^2^) [55]. Except for the study by Masanés et al. [26], all other studies used different BIA devices than Janssen et al. [56] (Table 2). Validity and precision of BIA results differ between manufacturers and depend on the hardware as well as the appropriate validation of the BIA-algorithm [119]. Discrepancies in the assumptions of the homogeneous bioelectrical model that lead to a higher measurement error occur with changes in hydration (e.g., edema) and with differences in body shape that are associated with aging (decreasing limb relative to trunk diameter), obesity (apple and pear shape of body fat distribution) and ethnicity (trunk to leg length, regional adiposity and muscularity). Therefore, segmental BIA that can measure the relative contribution of trunk and extremities to total body conductivity may help to reduce assumptions on body shape leading to an improved prediction compared with conventional wrist-ankle measurements [27]. The accuracy of phase-sensitive segmental BIA compared with MRI as a reference is clinically acceptable when whole body SM was assessed (two SDs: 11–12% for different ethnicities) but it was low when small compartments of the body were assessed (e.g., two SDs: 20–29% for the arms) [27]. 

### 4.1. Limitations of Proxies for Total Skeletal Muscle

Single SMA at L3 level turned out to be the best compromise site to assess volumes of total SM together with visceral adipose tissue (VAT) and subcutaneous adipose tissue (SAT) (*r* = 0.832–0.986; *p* < 0.01 [17]). Furthermore, SMA at L3 is considered as a valid proxy for whole body FFM (*r* = 0.940; *p* < 0.001 [120]). Other authors reported high correlations between single abdominal SMA at L4-L5 intervertebral space and total SM (*r* = 0.710–0.920 [121]), whereas the use of PMI to determine whole body SM is controversial because psoas is a relatively small muscle. A good correlation between PMI and SMI measured by BIA in healthy 35 Asian liver donors (*r* = 0.737; *p* < 0.001) and a moderate correlation in 137 living donor liver transplantation recipients (*r* = 0.682; *p* < 0.001) were found [63]. Other authors argue that L3 PMA is not representative of total SM [122,123]. Despite acceptable correlations, the accuracy of single images is limited in individual cases. Likewise, it is well established that the correlation between BMI and FM is fairly good at the population level whereas at the individual level BMI is only a poor indicator of adiposity [124]. In addition, validity of the assessment of changes in SM during follow-up is limited by the use of individual images from L3 or mid-thigh. These images cannot be used as pars pro toto because of regional differences in changes of muscle volume with age or obesity (e.g., the contribution of SM_MRI_ at the arms and legs to ASM tended to decrease at higher adiposity in both genders [104]). 

Similarly, ASM has limitations to assess the change in total SM with ageing or overweight and obesity. Since lean soft tissue from the extremities also contains lean compartments from connective tissue (e.g., skin and adipose tissue), SM accounts for only about 50% of FFM in obesity [116]. ASM was therefore shown to overestimate appendicular SM assessed by MRI with increasing BMI [27]. In line with this finding, DXA was also shown to underestimate the age-related loss of thigh muscle mass in comparison with MRI [125]. Furthermore, DXA measures of change in lean mass before and 10-week after resistance training were only modestly associated with MRI measures of change in muscle volume [126]. 

In summary, the random error of single images or ASM as a proxy for total SM limits the applicability of these substitutes in individual cases and together with the systematic error limit the accurate detection of changes in SM.

### 4.2. Normalization of Skeletal Muscle Mass for Body Size and Obesity

Normalization of lean mass for weight is inappropriate because two people with the same %FFM who differ in height have a different nutritional status, with the taller person having a lower muscularity [127]. FFM has been shown to scale to height with a power of around two in different ethnicities, ranging from 1.86 in non-Hispanic white women to 2.32 in non-Hispanic black men [128]. Consequently, appropriate normalization of total SM, SM-area, ASM and FFM is performed for height^2^.

In addition to the physiologic increase in SM with height, there is also an increase in SM with weight gain that depends on the initial amount of FM [129]. The evaluation of SM may thus also depend on the amount of FM. With increasing obesity, adverse effects on myocyte metabolism, muscle tissue composition and peak force generation can be mediated via paracrine signaling of proinflammatory immune cells in intermuscular adipose tissue [30]. The same SM at a higher FM may also lead to a limitation of strength and increased disability because at the same work load, energy expenditure and muscle force are higher for a person with obesity [130]. In line with these mechanisms, patients with a low SM and a concomitant high FM were shown to have a higher morbidity and mortality when compared to patients with a high FM only (for review see [131]). However, it remains unclear whether the risk of a low SM and a high FM is additive or if the risk of a high FM is disproportionally higher at a concomitantly low SM. 

Published definitions of sarcopenic obesity use BMI to assess overweight and obesity in combination with fixed cut-offs for a low SM that are derived from subjects with normal weight and/or overweight [72,76]. To the best of our knowledge, all current definitions disregard the relationship between fat and lean mass that can be investigated by applying the Forbes rule (energy partitioning, i.e., the fraction of energy lost or gained as protein, is a nonlinear function of FM [129]) or the Hattori chart (two dimensional plot of FMI vs. FFMI [132]). Table 5 provides novel BMI-dependent SMI cut-offs.

The combination of FFMI with FMI [133], %FM [6,8] or BMI [134] facilitate to investigate the proportional contribution of fat and lean compartments to health risk as well as their presumable interaction. An attractive alternative to the simultaneous use of two indices is integration of information on fat and lean compartments in one index as FM/FFM^2^. This index was proposed by Wells and Victoria who determined the appropriate power by which to raise the denominator from regressing FM on FFM [135]. The usefulness of this index needs to be investigated in future studies because it depends on a linear correlation between FM and FFM^2^, as well as on absence of heteroscedasticity. 

Beyond diverse methods of normalization (e.g., appendicular lean mass (ALM) adjusted by BMI [66,67], FFM normalized for body surface area (FFM_BSA_ = (weight [kg]^0.425^ × height [m]^0.725^) × 0.007184 [20])) heterogeneous outcome parameters (ASMI, SMI, L3 SMI, L3 PMI, FFMI) and a discrepant nomenclature for the same outcome parameter as well as different ways of reporting reference values hinder the comparison between studies. ASMI (i.e., appendicular skeletal muscle mass/height^2^) and SMI (total skeletal muscle mass/height^2^) were the most commonly used denominations within publications and therefore consistently applied in Table 1, Table 2, Table 3, Table 4 and Table 5. A great variety of different notations for the same outcome parameter were found for (a) SMI: e.g., skeletal muscle mass index, SMMI [52], muscle mass index, MMI [25,26], total skeletal muscle index, TSMI [53], total body skeletal muscle mass index, TBSMI [40] and also (b) ASMI: e.g., appendicular skeletal muscle mass index, ASMMI [136], appendicular muscle mass index, AMI (appendicular muscle mass (AMM)/height^2^) [54], relative appendicular skeletal muscle index, RASM [47,137], relative skeletal muscle mass index [138] and appendicular lean mass index (ALM/height^2^) [21]. In contrast to the heterogeneous nomenclature, some studies apply the same term “SMI” for different outcome parameters: e.g., ALM/BMI [66,67], ASM/height^2^ [46,139,140], ALM/height^2^ [141], ASM/body weight [53] and SM/body weight × 100 [25,137,142,143,144]. In cancer studies, SMI is normally defined as SMA/height^2^ [62,71,72]. Thus, a consistent nomenclature for proxies of SM is needed in order to facilitate comparison between studies.

Moreover, suitable reference values require an appropriate sample size ideally comprised of healthy or “normal” subjects (normative approach) or derive cut-offs from an older population or a group of patients (stratification approach). In addition, reference values can be reported using parametric methods, like Z-scores or 2 SDs below the mean, that rely on normal distribution of the data, on the absence of residual associations, and on constant variance of the normalized measurements throughout the entire sample (absence of heteroscedasticity, logarithmic transformation of the dependent variables or weighted regression models). In Table 1, Table 2, Table 3 and Table 4, most studies used cut-off thresholds for low SM on the basis of young healthy adults’ reference groups according to the recommendations proposed by the European Working Group on Sarcopenia in Older People [32]. The majority of these studies used two SDs below the means of healthy young subjects as a cut-off, e.g., [21,39,40,44,45,50] whereas other studies defined a low SM as one SD below the mean, e.g., [85,90,94,95]. Six articles stratified the cut-offs according to severity of a low SM [22,44,46,49,76,80]. One SM threshold was based on the fifth percentile [59] or on the 20th percentile [92] or on the 50th percentile [89]. Other studies used the sex-specific lowest quintiles [43], quartiles [47,62], tertiles [84], the lower two quintiles of the study population [98,100] or the lowest 20% of the distribution [38,42,48]. In one study, receiver operating characteristics analysis was used to develop SM cut-offs associated with physical disability [24]. In four studies, optimal stratification was used to determine the SM threshold of mortality risk in cancer patients [64,65,71,72]. Further diagnostic criteria applied classification and regression tree analysis [66,67].

## 5. Conclusions and Recommendations

In summary, published reference values for SM differ widely dependent on the outcome parameter and reference population. Results should consider the limitation of all proxies for total SM with respect to application in individual cases as well as for measurement of changes in SM. To facilitate comparison between results of different studies, authors should use a unified nomenclature for outcome parameters and indicate the device and software version of the body composition analyzer. In addition, the choice of body composition method should depend on the aim of the study. For assessment of changes in SM and evaluation of individual patients, a high precision is required that is, for instance, not fulfilled when segmental bioelectrical impedance is used to assess limb SM. The adverse effects of obesity on muscle quality and function may lead to an underestimation of sarcopenia in obesity and therefore requires normalization of SM for FM.

## Figures and Tables

**Table 1:** Cut-off values and diagnostic criteria of a low muscle mass using dual X-ray absorptiometry (DXA).

Reference	Device/Software	Parameter/Cut-Off by Gender	Reference Group Characteristics (Mean ± SD)/*Diagnostic Criteria (→)*		
Alkahtani (2017)	Lunar iDXA General Electric machine, Healthcare	ASMI Class I and Class II sarcopenia men: 7.74 kg/m^2^ and 6.51 kg/m^2^	*n* = 232	Saudi Arabians	
				men	women
			*n*	232	0
			Age (y)	27.1 ± 4.2	
			BMI (kg/m^2^)	28.1 ± 5.5	
			*→ Class I sarcopenia: 1 SD below the means for young, healthy adults* *→ Class II sarcopenia: 2 SDs below the means for young, healthy adults*		
Imboden et al. (2017)	GE Lunar Prodigy or iDXA	(a) ASMI men: 6.35 kg/m^2^ women: 4.92 kg/m^2^	(a) *n* = 1246	US population	
				men	women
			*n*	488	758
			Age (y)	20 to 39	20 to 39
			BMI (kg/m^2^)	NA	NA
			*→ 2 SDs below the sex-specific means of young adults*		
		(b) ASMI men: 7.40 kg/m^2^ women: 5.60 kg/m^2^	(b) *n* = 351	US population	
				men	women
			*n*	168	183
			Age (year)	70 to 79	70 to 79
			BMI (kg/m^2^)	NA	NA
			*→ sex-specific lowest 20% of study group*		
Kruger et al. (2015)	Hologic Discovery-W, software version 12.7 for Cape Town QDR-4500A, software version 12.5:7 for Soweto	(a) ASMI women: 4.93 kg/m^2^	(a) *n* = 238	Black South Africans (Cape Town)	
				men	women
			*n*	0	238
			Age (year)		25.8 ± 5.9
			BMI (kg/m^2^)		29.8 ± 8.0
			*→ 2 SDs below the sex-specific means of young, healthy adults*		
		(b) ASMI women: 4.95 kg/m^2^			
			(b) *n* = 371	Black South Africans (Soweto)	
				men	women
			*n*	0	371
			Age (year)		35.1 ± 3.2
			BMI (kg/m^2^)		28.8 ± 6.2
			*→ 2 SDs below the sex-specific means of young, healthy adults*		
Alemán-Mateo & Ruiz Valenzuela (2014)	DPX-MD+, GE Lunar	ASMI men: 5.86 kg/m^2^ women: 4.72 kg/m^2^ SMI men: 6.63 kg/m^2^ women: 5.22 kg/m^2^ SM was predicted using Kim’s equation (Kim et al., 2002)	*n* = 216	Mexicans	
				men	women
			*n*	136	80
			Age (year)	27.3 ± 5.0	28.2 ± 5.6
			BMI (kg/m^2^)	25.7 ± 3.6	23.2 ± 3.1
			*→ 2 SDs below the sex-specific means of young, healthy adults*		
Gould et al. (2014)	DPX-L scanner, software version 1.31; Lunar or Prodigy Pro, Lunar	ASMI men: 6.94 kg/m^2^ women: 5.30 kg/m^2^	*n* = 682	study performed in southeastern Australia	
				men	women
			*n*	374	308
			Age (year)	20 to 39	20 to 39
			BMI (kg/m^2^)	NA	NA
			*→ 2 SDs below the sex-specific means of young adults*		
Marwaha et al. (2014)	Prodigy Oracle, GE Lunar Corp.	(a) ASMI women: 4.42 kg/m^2^	(a) *n* = 469	Indians	
				men	women
			*n*	0	469
			Age (year)		20 to 39
			BMI (kg/m^2^)		NA
			*→ 2 SDs below the sex-specific means of young adults*		
		(b) ASMI women: 5.11 kg/m^2^	(b) *n* = 1045	Indians	
				men	women
			*n*	0	1045
			Age (year)		44.0 ± 17.1
			BMI (kg/m^2^)		25.0 ± 5.2
			*→ sex-specific lowest 20% of study group*		
Yu et al. (2014)	Hologic Delphi W4500 densitometer, auto whole body version 12.4	ASMI men: 6.52 kg/m^2^ women: 5.44 kg/m^2^	*n* = 4000	Chinese (Hong Kong)	
				men	women
			*n*	2000	2000
			Age (year)	72.5 ± 5.2	72.5 ± 5.2
			BMI (kg/m^2^)	23.7 ± 3.3	23.7 ± 3.3
			*→ lowest quintile*		
Kim et al. (2012)	Hologic Discovery-W	ASMI Class I and Class II sarcopenia men: 7.50 kg/m^2^ and 6.58 kg/m^2^ women: 5.38 kg/m^2^ and 4.59 kg/m^2^	*n* = 2513	Koreans	
				men	women
			*n*	1245	1268
			Age (year)	31.0 ± 5.5	30.8 ± 5.6
			BMI (kg/m^2^)	24.0 ± 3.4	22.1 ± 3.5
			*→ Class I sarcopenia: 1-2 SDs below the sex-specific means for young, healthy adults* *→ Class II sarcopenia: 2 SDs below the sex-specific means for young, healthy adults*		
Oliveira et al. (2011)	DPX-L, Lunar Radiation Corporation	ASMI women: 5.0 kg/m^2^	*n* = 349	Brazilians	
				men	women
			*n*	0	349
			Age (year)		29.0 ± 7.5
			BMI (kg/m^2^)		23.5 ± 4.5
			*→ 2 SDs below the sex-specific means of young, healthy adults*		
Sanada et al. (2010)	Hologic QDR-4500A scanner, software version 11.2:3	ASMI Class I and Class II sarcopenia men: 7.77 kg/m^2^ and 6.87 kg/m^2^ women: 6.12 kg/m^2^ and 5.46 kg/m^2^	*n* = 529	Japanese	
				men	women
			*n*	266	263
			Age (year)	28.2 ± 7.4	28.0 ± 7.0
			BMI (kg/m^2^)	23.0 ± 3.0	20.8 ± 2.6
			*→ Class I sarcopenia: 1 SD below the sex-specific means for young, healthy adults* *→ Class II sarcopenia: 2 SDs below the sex-specific means for young, healthy adults*		
Szulc et al. (2004)	Hologic 1000W	ASMI men: 6.32 kg/m^2^	*n* = 845	study performed in France	
				men	women
			*n*	845	0
			Age (year)	64.0 ± 8.0	
			BMI (kg/m^2^)	28.0 ± 3.7	
			*→ lowest quartile*		
Newman et al. (2003)	QDR 4500A, Hologic, Inc.	ASMI men: 7.23 kg/m^2^ women: 5.67 kg/m^2^ Values recommended by the International Working Group on Sarcopenia (Fielding et al., 2011)	*n* = 2984	study performed in USA (41% Blacks)	
				men	women
			*n*	1435	1549
			Age (year)	73.6 ± 2.9	73.6 ± 2.9
			BMI (kg/m^2^)	27.4 ± 4.8	27.4 ± 4.8
			*→ sex-specific lowest 20% of study group*		
Tankó et al. (2002)	QDR4500A scanner, Hologic, software version V8.10a:3 and DPX scanner, Lunar Radiation, software versions 3.1 and 3.2	(a) ASMI women: 6.10 kg/m^2^ (b) ASMI women: 5.40 kg/m^2^	*n* = 216 women	Danes	
				men	women
			*n*	0	216
			Age (year)		30.4 ± 5.3
			BMI (kg/m^2^)		NA
			*→ (a) 1-2 SDs below the sex-specific means for young, healthy, premenopausal women* *→ (b) 2 SDs below the sex-specific means for young, healthy, premenopausal women*		
Baumgartner et al. (1998)	Lunar DPX	ASMI men: 7.26 kg/m^2^ women: 5.45 kg/m^2^	*n* = 229	US population (non-Hispanic white men and women)	
				men	women
			*n*	107	122
			Age (year)	28.7 ± 5.1	29.7 ± 5.9
			BMI (kg/m^2^)	24.6 ± 3.8	24.1 ± 5.4
			*→ 2 SDs below the sex-specific means of young, healthy adults*		

ASMI, appendicular skeletal muscle mass index; BMI, body mass index; DXA, dual X-ray absorptiometry; NA, not available; SD, standard deviation; SM, skeletal muscle mass; SMI, skeletal muscle mass index.

**Table 2:** Cut-off values and diagnostic criteria of a low muscle mass using bioelectrical impedance analysis (BIA).

Reference	Device/Software	Parameter/Cut-Off by Gender	Reference Group Characteristics (Mean ± SD)/*Diagnostic Criteria (→)*		
Krzymińska-Siemaszko et al. (2019)	InBody 170 analyzer, Biospace Co.	ASMI men: 7.35 kg/m^2^ (20–30 y), 7.38 kg/m^2^ (18–40 y, 18–39 y, 20–35 y), 7.40 kg/m^2^ (20–39 y, 20–40 y) women: 5.51 kg/m^2^ (20–30 y), 5.56 kg/m^2^ (18–40 y), 5.53 kg/m^2^ (18–39 y), 5.59 kg/m^2^ (20–39 y), 5.60 kg/m^2^ (20–40 y), 5.58 kg/m^2^ (20–35 y) Authors recommended the highest cut-off points, i.e., 5.60 kg/m^2^ in women and 7.40 kg/m^2^ in men	*n* = 1512	study performed in Poland (Caucasians)	
				men	women
			*n*	635	877
			Age (year)	24.2 ± 5.3	28.4 ± 6.8
			BMI (kg/m^2^)	NA	NA
			total *n* for men and women depends on age range		
			*→ 2 SDs below the sex-specific means of young, healthy adults*		
Alkahtani (2017)	Tanita MC-980MA, Tanita Corporation Inbody 770, Inbody Co.	ASMI Class I and Class II sarcopenia men: 8.68 kg/m^2^ and 7.45 kg/m^2^ ASMI Class I and Class II sarcopenia men: 7.29 kg/m^2^ and 6.42 kg/m^2^	*n* = 232	Saudi Arabians	
				men	women
			*n*	232	0
			Age (year)	27.1 ± 4.2	
			BMI (kg/m^2^)	28.1 ± 5.5	
			*→ Class I sarcopenia: 1 SD below the means for young, healthy adults* *→ Class II sarcopenia: 2 SDs below the means for young, healthy adults*		
Bahat et al. (2016)	Tanita BC 532 model body analysis monitor	SMI men: 9.2 kg/m^2^ women: 7.4 kg/m^2^ SM (kg) = 0.566 x FFM	*n* = 301	study performed in Turkey	
				men	women
			*n*	187	114
			Age (year)	26.8 ± 4.5	25.9 ± 4.7
			BMI (kg/m^2^)	25.5 ± 3.6	22.4 ± 3.4
			*→ 2 SDs below the sex-specific means of young, healthy adults*		
Chang et al. (2013)	Tanita BC-418	ASMI men: 6.76 kg/m^2^ women: 5.28 kg/m^2^ SMI men: 7.70 kg/m^2^ women: 5.67 kg/m^2^ SM by Janssen et al. (2000) equation	*n* = 998	Taiwanese	
				men	women
			*n*	498	500
			Age (year)	23.1 ± 3.0	23.1 ± 2.7
			BMI (kg/m^2^)	22.2 ± 3.1	20.2 ± 2.6
			*→ 2 SDs below the sex-specific means of young, healthy adults*		
Yamada et al. (2013)	Inbody 720, Biospace Co.	ASMI men: 6.75 kg/m^2^ women: 5.07 kg/m^2^	*n* = 38,099	Japanese	
				men	women
			*n*	19,797	18,302
			Age (year)	18 to 40	18 to 40
			BMI (kg/m^2^)	NA	NA
			*→ 2 SDs below the sex-specific means of young adults*		
Masanés et al. (2012)	RJL Systems BIA 101	SMI men: 8.25 kg/m^2^ women: 6.68 kg/m^2^ SM by Janssen et al. (2000) equation	*n* = 230	study performed in Spain	
				men	women
			*n*	110	120
			Age (year)	28.6 ± 5.0	28.2 ± 6.0
			BMI (kg/m^2^)	24.6 ± 2.6	21.9 ± 2.2
			*→ 2 SDs below the sex-specific means of young, healthy adults*		
Tanimoto et al. (2012)	Tanita MC-190	ASMI men: 7.0 kg/m^2^ women: 5.8 kg/m^2^	*n* = 1719	Japanese	
				men	women
			*n*	838	881
			Age (year)	26.6 ± 6.7	28.5 ± 7.3
			BMI (kg/m^2^)	22.4 ± 3.2	20.8 ± 2.9
			*→ 2 SDs below the sex-specific means of young, healthy adults*		
Chien et al. (2008)	Maltron BioScan 920	SMI men: 8.87 kg/m^2^ women: 6.42 kg/m^2^ SM by Janssen et al. (2000) equation	*n* = 200	Taiwanese	
				men	women
			*n*	100	100
			Age (year)	26.7 ± 5.7	27.6 ± 5.9
			BMI (kg/m^2^)	23.2 ± 3.5	20.6 ± 2.5
			*→ 2 SDs or more below the sex-specific means of young, healthy adults*		
Tichet et al. (2008)	Impedimed multifrequency analyser	SMI men: 8.60 kg/m^2^ women: 6.20 kg/m^2^ SM by Janssen et al. (2000) equation	*n* = 782	French people	
				men	women
			*n*	394	388
			Age (year)	30.2 ± 6.1	29.2 ± 6.3
			BMI (kg/m^2^)	23.9 ± 3.0	22.5 ± 3.4
			*→ 2 SDs below the sex-specific means of young, healthy adults*		
Janssen et al. (2004)	Valhalla 1990B Bio-Resistance Body Composition Analyzer	SMI moderate and severe sarcopenia men: 8.51–10.75 kg/m^2^ and ≤8.50 kg/m^2^ women: 5.76–6.75 kg/m^2^ and ≤5.75 kg/m^2^ SM by Janssen et al. (2000) equation	*n* = 4499	US population (non-Hispanic White, non-Hispanic Black and Mexican American)	
				men	women
			*n*	2223	2276
			Age (year)	70.0 ± 7.0	71.0 ± 8.0
			BMI (kg/m^2^)	26.6 ± 4.3	27.0 ± 5.5
			*→ receiver operating characteristics*		

ASMI, appendicular skeletal muscle mass index; BIA, bioelectrical impedance analysis; BMI, body mass index; FFM, fat-free mass; NA, not available; SD, standard deviation; SM, skeletal muscle mass; SMI, skeletal muscle mass index.

**Table 3:** Cut-off values and diagnostic criteria of a low muscle mass using computed tomography (CT).

Reference	Device/Software	Parameter/Cut-Off by Gender	Reference Group Characteristics (Mean ± SD)/*Diagnostic Criteria (→)*		
Ufuk & Herek (2019)	lumbar CT images (16-detector row, Brilliance)	CT L3 SMI men: 44.98 cm^2^/m^2^ women: 36.05 cm^2^/m^2^ CT L3 PMI men: 2.63 cm^2^/m^2^ women: 2.02 cm^2^/m^2^	*n* = 270	healthy Turkish population	
				men	women
			*n*	134	136
			Age (year)	44.3 ± 11.2	45.0 ± 8.6
			BMI (kg/m^2^)	26.4 ± 3.5	25.4 ± 3.6
			*→ 2 SDs below the sex-specific means of young adults*		
Derstine et al. (2018)	lumbar CT images (GE Discovery or LightSpeed scanner)	(a) CT L3 SMI men: 45.4 cm^2^/m^2^ women: 34.4 cm^2^/m^2^	(a) *n* = 727	healthy US population	
				men	women
			*n*	317	410
			Age (year)	18 to 40	18 to 40
			BMI (kg/m^2^)	NA	NA
			*→ 2 SDs below the sex-specific means of young adults*		
		(b) CT T10 SMI men: 28.8 cm^2^/m^2^ women: 20.4 cm^2^/m^2^	(b) *n* = 278	healthy US population	
				men	women
			*n*	122	156
			Age (year)	18 to 40	18 to 40
			BMI (kg/m^2^)	NA	NA
			*→ 2 SDs below the sex-specific means of young adults*		
		(c) CT T11 SMI men: 27.6 cm^2^/m^2^ women: 19.2 cm^2^/m^2^	(c) *n* = 577	healthy US population	
				men	women
			*n*	241	366
			Age (year)	18 to 40	18 to 40
			BMI (kg/m^2^)	NA	NA
			*→ 2 SDs below the sex-specific means of young adults*		
		(d) CT T12 SMI men: 28.8 cm^2^/m^2^ women: 20.8 cm^2^/m^2^	(d) *n* = 700	healthy US population	
				men	women
			*n*	299	401
			Age (year)	18 to 40	18 to 40
			BMI (kg/m^2^)	NA	NA
			*→ 2 SDs below the sex-specific means of young adults*		
		(e) CT L1 SMI men: 34.6 cm^2^/m^2^ women: 25.9 cm^2^/m^2^	(e) *n* = 724	healthy US population	
				men	women
			*n*	315	409
			Age (year)	18 to 40	18 to 40
			BMI (kg/m^2^)	NA	NA
			*→ 2 SDs below the sex-specific means of young adults*		
		(f) CT L2 SMI men: 40.1 cm^2^/m^2^ women: 30.4 cm^2^/m^2^	(f) *n* = 726	healthy US population	
				men	women
			*n*	315	411
			Age (year)	18 to 40	18 to 40
			BMI (kg/m^2^)	NA	NA
			*→ 2 SDs below the sex-specific means of young adults*		
		(g) CT L4 SMI men: 41.3 cm^2^/m^2^ women: 34.2 cm^2^/m^2^	(g) *n* = 704	healthy US population	
				men	women
			*n*	305	399
			Age (year)	18 to 40	18 to 40
			BMI (kg/m^2^)	NA	NA
			*→ 2 SDs below the sex-specific means of young adults*		
		(h) CT L5 SMI men: 39.0 cm^2^/m^2^ women: 30.6 cm^2^/m^2^	(h) *n* = 506	healthy US population	
				men	women
			*n*	211	295
			Age (year)	18 to 40	18 to 40
			BMI (kg/m^2^)	NA	NA
			*→ 2 SDs below the sex-specific means of young adults*		
van der Werf et al. (2018)	lumbar CT images (64-row CT scanner, Sensation 64, Siemens or CT Brilliance 64, Philips)	CT L3 SMI men: 44.6 cm^2^/m^2^ women: 34.0 cm^2^/m^2^	*n* = 300	healthy Caucasian population	
				men	women
			*n*	126	174
			Age (y)	20 to 60	20 to 60
			BMI (kg/m^2^)	NA	NA
			*→ 5th percentile*		
Benjamin et al. (2017)	lumbar CT images (Discovery 750 HD 64-row spectral CT scanner)	CT L3 SMI men: 36.54 cm^2^/m^2^ women: 30.21 cm^2^/m^2^	*n* = 275	healthy Asian Indians	
				men	women
			*n*	139	136
			Age (year)	32.2 ± 9.8	32.2 ± 9.8
			BMI (kg/m^2^)	24.2 ± 3.2	24.2 ± 3.2
			*→ 2 SDs below the sex-specific means of young adults*		
Kim et al. (2017)	lumbar CT images (64-slice multidetector CT scanner, Brilliance 64, Philips Healthcare)	CT L3 PMI men: 5.92 cm^2^/m^2^ (20–39 y), 4.74 cm^2^/m^2^ (40–49 y), 4.22 cm^2^/m^2^ (50–59 y), 3.74 cm^2^/m^2^ (60–69 y), 3.32 cm^2^/m^2^ (70–89 y) women: 4.0 cm^2^/m^2^ (20–39 y), 2.88 cm^2^/m^2^ (40–49 y), 2.43 cm^2^/m^2^ (50–59 y), 2.20 cm^2^/m^2^ (60–69 y), 1.48 cm^2^/m^2^ (70–89 y)	*n* = 1422	study performed in Korea	
				men	women
			*n*	550	872
			Age (year)	52.4 ± 12.0	53.3 ± 12.2
			BMI (kg/m^2^)	24.5 ± 3.1	22.8 ± 3.2
			total n for men and women depends on age range		
			*→ 2 SDs below the sex-specific means of young, healthy adults*		
Sakurai et al. (2017)	lumbar CT images	CT L3 SMI men: 43.2 cm^2^/m^2^ women: 34.6 cm^2^/m^2^	*n* = 569 patients with gastric cancer	study performed in Japan	
				men	women
			*n*	396	173
			Age (year)	66.7 ± 11.2	66.7 ± 11.2
			BMI (kg/m^2^)	22.0 ± 3.4	22.0 ± 3.4
			*→ lowest sex-specific quartile*		
Hamaguchi et al. (2016)	lumbar CT images (Aquilion 64, Toshiba Medical Systems)	CT L3 PMI men: 6.36 cm^2^/m^2^ women: 3.92 cm^2^/m^2^	*n* = 230	healthy Asian population	
				men	women
			*n*	116	114
			Age (year)	20 to 49	20 to 49
			BMI (kg/m^2^)	NA	NA
			*→ 2 SDs below the sex-specific means of young adults*		
Zhuang et al. (2016)	lumbar CT images	CT L3 SMI men: 40.8 cm^2^/m^2^ women: 34.9 cm^2^/m^2^	*n* = 937 patients with gastric cancer	study performed in China	
				men	women
			*n*	730	207
			Age (year)	64.0 ± 15.0	64.0 ± 15.0
			BMI (kg/m^2^)	21.9 ± 3.0	21.9 ± 3.0
			*→ optimal stratification*		
Iritani et al. (2015)	lumbar CT images	CT L3 SMI men: 36.0 cm^2^/m^2^ women: 29.0 cm^2^/m^2^	*n* = 217 patients with hepatocellular carcinoma	study performed in Japan	
				men	women
			*n*	146	71
			Age (year)	27 to 90	27 to 90
			BMI (kg/m^2^)	13.4 to 35.9	13.4 to 35.9
			*→ optimal stratification*		

BMI, body mass index; CT, computed tomography; L, lumbar vertebra; L3, third lumbar vertebra; NA, not available; PMI, psoas muscle index; SD, standard deviation; SMI, skeletal muscle mass index; T, thoracic vertebra.

**Table 4:** Cut-off values that combine measures of muscle mass and obesity.

Reference	Device/Software	Parameter/Cut-Off by Gender	Reference Group Characteristics (Mean ± SD)/*Diagnostic Criteria (→)*		
Prado et al. (2008)	CT images	CT L3 SMI: men: ≤52.4 cm^2^/m^2^ women: ≤38.5 cm^2^/m^2^ + BMI ≥ 30 kg/m^2^	*n* = 250 obese patients with cancers of the respiratory tract and gastrointestinal locations	study performed in Canada	
				men	women
			*n*	136	114
			Age (year)	64.6 ± 10.2	63.2 ± 10.5
			BMI (kg/m^2^)	33.9 ± 4.4	34.7 ± 4.3
			*→ optimal stratification*		
Martin et al. (2013)	CT images	CT L3 SMI: men: <43 cm^2^/m^2^ women: <41 cm^2^/m^2^ for BMI < 25 kg/m^2^ men: <53 cm^2^/m^2^ for BMI ≥ 25 kg/m^2^	*n* = 1473 patients with cancers of the respiratory tract and gastrointestinal locations	study performed in Canada	
				men	women
			*n*	828	645
			Age (year)	64.7 ± 11.2	64.8 ± 11.5
			BMI (kg/m^2^)	26.0 ± 4.9	25.1 ± 5.8
			*→ optimal stratification*		
Muscariello et al. (2016)	BIA (RJL 101, Akern SRL)	(a) SMI + BMI < 25 kg/m^2^ Class I and Class II sarcopenia women: 7.4 and 6.8 kg/m^2^	(a) *n* = 313	study performed in Italy	
				men	women
			*n*	0	313
			Age (year)		28.5 ± 7.6
			BMI (kg/m^2^)		24.1 ± 2.5
			*→ Class I sarcopenia: 1 SD below the sex-specific means of young adults*		
			*→ Class II sarcopenia: 2 SDs below the sex-specific means of young adults*		
		(b) SMI + BMI ≥ 30 kg/m^2^ Class I and Class II sarcopenia women: 8.3 and 7.3 kg/m^2^ SM by Janssen et al. (2000) equation	(b) *n* = 361	study performed in Italy	
				men	women
			*n*	0	361
			Age (year)		30.9 ± 7.9
			BMI (kg/m^2^)		35.1 ± 4.6
			*→ Class I sarcopenia: 1 SD below the sex-specific means of young adults*		
			*→ Class II sarcopenia: 2 SDs below the sex-specific means of young adults*		
Nishigori et al. (2016)	CT images	CT L3 SMI (Prado et al. 2008): men: ≤52.4 cm^2^/m^2^ women: ≤38.5 cm^2^/m^2^ + visceral fat area (VFA) ≥100 cm^2^ in both sexes	reference group characteristic CT L3 SMI see Prado et al. (2008)		
Pecorelli et al. (2016)	CT images	(a) CT L3 SMI (Prado et al. 2008): men: ≤52.4 cm^2^/m^2^ women: ≤38.5 cm^2^/m^2^ + (b) visceral fat area/total abdominal muscle area ratio (VFA/TAMA) men & women: 3.2	(a) reference group characteristic CT L3 SMI see Prado et al. (2008)		
			(b) *n* = 202 patients with resectable pancreas, periampullary	study performed in Italy	
				men	women
			*n*	108	94
			Age (year)	66.8 ± 10.7	66.8 ± 10.7
			BMI (kg/m^2^)	23.6 ± 3.7	23.6 ± 3.7
			*→ optimal stratification*		
Kwon et al. (2017)	DXA (Discovery QDR 4500, Hologic)	ASM (as % of body weight) men: 30.98% women: 24.81% + BMI ≥ 25 kg/m^2^ (based on the definition in the Asian-Pacific region)	*n* = 3550	Koreans	
				men	women
			*n*	1668	1882
			Age (year)	20 to 39	20 to 39
			BMI (kg/m^2^)	NA	NA
			*→ 1 SD below the sex-specific means of young adults*		
Chiles Shaffer et al. (2017)	DXA (Lunar Prodigy Advance with GE EnCore 2006 version 10.51.0006)	ASM adjusted for BMI men: <0.725 kg/m^2^ women: <0.591 kg/m^2^	*n* = 545	study performed in US	
				men	women
			*n*	287	258
			Age (year)	79.2 ± 7.2	77.7 ± 7.3
			BMI (kg/m^2^)	27.2 ± 3.8	27.0 ± 5.2
			*→ CART analysis*		
An & Kim (2016)	DXA (Discovery-W, Hologic)	ASM (as % of body weight) men: 30.1% women: 21.2% + WC ≥ 90 cm in men WC ≥ 80 cm in women (sex-specific cut-off for Asians)	*n* = 5944	study performed in Korea	
				men	women
			*n*	2502	3334
			Age (year)	20 to 39	20 to 39
			BMI (kg/m^2^)	NA	NA
			*→ 1 SD below the sex-specific means of young adults*		
Cho et al. (2015)	(a) DXA (Discovery-W, Hologic)	(a) ASM (as % of body weight) men: 30.3% women: 23.8% + WC ≥ 90 cm in men WC ≥ 85 cm in women	(a) *n* = 4987	Koreans	
				men	women
			*n*	2123	2864
			Age (year)	20 to 39	20 to 39
			BMI (kg/m^2^)	NA	NA
			*→ 1 SD below the sex-specific means of young, healthy adults*		
Oh et al. (2015)	DXA (Lunar Corp.)	ASM (as % of body weight) men: 44% women: 52% + BMI ≥ 25 kg/m^2^	*n* = 1746	Koreans	
				men	women
			*n*	748	998
			Age (year)	20 to 39	20 to 39
			BMI (kg/m^2^)	NA	NA
			*→ 1 SD below the sex-specific means of young, healthy adults*		
Lee et al. (2015)	DXA (Discovery QDR 4500, Hologic)	ASM (as % of body weight) men: 32.2% women: 25.5% + BMI ≥ 25 kg/m^2^ (based on the criteria of the Asian-Pacific region)	*n* = 2200	Koreans	
				men	women
			*n*	960	1240
			Age (year)	20 to 30	20 to 30
			BMI (kg/m^2^)	NA	NA
			*→ 1 SD below the sex-specific means of young, healthy adults*		
Baek et al. (2014)	DXA (Lunar Corp.)	ASMI men: 6.96 kg/m^2^ women: 4.96 kg/m^2^ ASM (as % of body weight) men: 30.65% women: 23.90% + BMI ≥ 25 kg/m^2^ (IOTF-proposed classification of BMI for Asia)	*n* = 4192	Koreans	
				men	women
			*n*	1699	2493
			Age (year)	20 to 39	20 to 39
			BMI (kg/m^2^)	NA	NA
			*→ 1 SD below the sex-specific means of young, healthy adults*		
Cawthon et al. (2014)	DXA (QDR 4500, Hologic 2000, Lunar Prodigy)	ASM adjusted for BMI men: <0.789 women: <0.512 recommended by FNIH (Studenski et al., 2014)	*n* = 11,270	study performed in US	
				men	women
			*n*	7582	3688
			Age (year)	65 to 80	65 to 80
			BMI (kg/m^2^)	NA	NA
			*→ CART analysis plus sensitivity analyses*		
Chung et al. (2013)	(a) DXA (fan-beam technology, Lunar Corp.)	(a) ASM (as % of body weight) men: 32.5% women: 25.7% + BMI ≥ 25 kg/m^2^ (IOTF-proposed classification of BMI for Asia)	(a) *n* = 2781	study performed in Korea	
				men	women
			*n*	1155	1626
			Age (year)	20 to 39	20 to 39
			BMI (kg/m^2^)	NA	NA
			*→ 1 SD below the sex-specific means of young, healthy adults*		
Hwang et al. (2012)	DXA (Discovery-W, Hologic)	ASM (as % of body weight) men: 29.53%women: 23.20% + WC ≥ 90 cm in men WC ≥ 85 cm in women (Korean abdominal obesity criteria; Lee et al., 2007)	*n* = 2269	Koreans	
				men	women
			*n*	1003	1266
			Age (year)	30.7 ± 5.5	31.0 ± 5.5
			BMI (kg/m^2^)	24.1 ± 3.5	22.1 ± 3.6
			*→ 2 SDs below the sex-specific means of young adults*		
Lee et al. (2012)	DXA (Discovery-W, Hologic)	ASM (as % of body weight) men: 26.8% women: 21.0% + BMI ≥ 27.5 kg/m^2^	*n* = 2113	Koreans	
				men	women
			*n*	902	1211
			Age (year)	20 to 40	20 to 40
			BMI (kg/m^2^)	NA	NA
			*→ 2 SDs below the sex-specific means of young, healthy adults*		
Kim et al. (2012)	DXA (Discovery-W, Hologic)	ASM (as % of body weight) Class II sarcopenia men: 29.1% women: 23.0% ASMI Class II sarcopenia men: 6.58 kg/m^2^ women: 4.59 kg/m^2^ + WC ≥ 90 cm in men (Lee et al., 2007) WC ≥ 85 cm in women	*n* = 2513	Koreans	
				men	women
			*n*	1245	1268
			Age (year)	31.0 ± 5.5	30.8 ± 5.6
			BMI (kg/m^2^)	24.0 ± 3.4	22.1 ± 3.5
			*→ 2 SDs below the sex-specific means of young, healthy adults*		
Kim et al. (2011)	DXA (Lunar Corp.)	ASM (as % of body weight) men: 29.5% women: 23.2% + BMI ≥ 27.5 kg/m^2^	*n* = 2392	study performed in Korea	
				men	women
			*n*	1054	1338
			Age (year)	20 to 40	20 to 40
			BMI (kg/m^2^)	NA	NA
			*→ 2 SDs below the sex-specific means of young, healthy adults*		
Kim et al. (2009)	DXA (Discovery A, Hologic)	(a) ASMI men: 8.81 kg/m^2^ women: 7.36 kg/m^2^ + (b) FM men: 20.21% women: 31.71%	*n* = 526	Koreans	
				men	women
			*n*	198	328
			Age (year)	52.2 ± 14.4	51.2 ± 14.8
			BMI (kg/m^2^)	25.2 ± 3.1	23.9 ± 3.7
			*→ (a) lower two quintiles*		
			*→ (b) two highest quintiles*		
Rolland et al. (2009)	(a) DXA (Lunar DPX, Lunar Corp.)	(a) ASMI women: 5.45 kg/m^2^ (Baumgartner et al., 1998) +	(a) *n* = 122	US population (non-Hispanic white men and women)	
				men	women
			*n*	0	122
			Age (year)		29.7 ± 5.9
			BMI (kg/m^2^)		24.1 ± 5.4
			*→ 2 SDs below the sex-specific means of young, healthy adults*		
	(b) DXA (QDR 4500 W, Hologic)	(b) FM women: 40%	(b) *n* = 1308	study performed in France	
				men	women
			*n*	0	1308
			Age (year)		≥75
			BMI (kg/m^2^)		NA
			*→ 60th percentile of the healthy study sample*		
Baumgartner et al. (1998)	DXA (Lunar DPX, Lunar Corp.)	(a) ASMI men: 7.26 kg/m^2^ women: 5.45 kg/m^2^ + (b) FM men: 27% women: 38%	*n* = 229	US population (non-Hispanic white men and women)	
				men	women
			*n*	107	122
			Age (year)	28.7 ± 5.1	29.7 ± 5.9
			BMI (kg/m^2^)	24.6 ± 3.8	24.1 ± 5.4
			*(a) → 2SDs below the sex-specific means of young, healthy adults* *(b)* → >sex-specific median		
Bahat et al. (2016); Bahat et al. (2018)	BIA (Tanita-BC532)	(a) SMI men: 9.2 kg/m^2^ women: 7.4 kg/m^2^ SM (kg) = 0.566 × FFM +	(a) *n* = 301	study performed in Turkey	
				men	women
			*n*	187	114
			Age (year)	26.8 ± 4.5	25.9 ± 4.7
			BMI (kg/m^2^)	25.5 ± 3.6	22.4 ± 3.4
			*→ 2 SDs below the sex-specific means of young, healthy adults*		
		
		(b) FM men: 27.3% women: 40.7%	(b) *n* = 992	study performed in Turkey	
				men	women
			*n*	308	684
			Age (year)	75.2 ± 7.2	75.2 ± 7.2
			BMI (kg/m^2^)	27.7 ± 4.3	30.7 ± 5.6
			*→ above 60th percentile*		
Ishii et al. (2016)	(a) BIA (Tanita MC-190)	(a) ASMI men: 7.0 kg/m^2^ women: 5.8 kg/m^2^ +	(a) *n* = 1719	Japanese	
				men	women
			*n*	838	881
			Age (year)	26.6 ± 6.7	28.5 ± 7.3
			BMI (kg/m^2^)	22.4 ± 3.2	20.8 ± 2.9
			*→ 2 SDs below the sex-specific means of young, healthy adults*		
	(b) BIA (InBody 430, Biospace)	(b) FM men: 29.7% women: 37.2%	(b) *n* = 1731	Japanese	
				men	women
			*n*	875	856
			Age (year)	≥ 65	≥ 65
			BMI (kg/m^2^)	NA	NA
			*→ highest quintile*		
Moreira et al. (2016)	BIA (InBody R20, Biospace)	ASMI women: 6.08 kg/m^2^ + WC ≥ 88 cm in women (Brazilian obesity guidelines)	*n* = 491	study performed in Northeast Brazil (Whites, Blacks, Pardo)	
				men	women
			*n*	0	491
			Age (year)		50.0 ± 5.6
			BMI (kg/m^2^)		29.0 ± 4.8
			*→ 20th percentile*		
Kemmler et al. (2016)	BIA (InBody 770, Biospace)	(a) ASMI women: 5.66 kg/m^2^	(a) *n* = 689	study performed in Germany (Caucasians)	
				men	women
			*n*	0	689
			Age (year)		18 to 35
			BMI (kg/m^2^)		NA
			*→ 2 SDs below the sex-specific means of young, healthy adults*		
		(b) ASMI women: 5.99 kg/m^2^ + BMI ≥ 30 kg/m^2^ (NIH) FM ≥ 35% (WHO)	(b) *n* = 1325	study performed in Germany (Caucasians)	
				men	women
			*n*	0	1325
			Age (year)		76.4 ± 4.9
			BMI (kg/m^2^)		26.7 ± 4.3
			*→ lowest quintile*		
Lee et al. (2016)	BIA (InBody 720, Biospace)	(a) SMI (as % of body weight) men: 38.2 % women: 32.2% SM by Janssen et al. (2000) equation +	(a) *n* = 273	study performed in Korea	
				men	women
			*n*	157	116
			Age (year)	25.5 ± 2.9	26.1 ± 4.6
			BMI (kg/m^2^)	24.1 ± 3.0	20.7 ± 2.6
			*→ 2 SDs below the sex-specific means of young, healthy adults*		
		(b) FM men: 25.8% women: 36.5%	(b) *n* = 309	study performed in Korea	
				men	women
			*n*	85	224
			Age (year)	70.7 ± 6.3	66.4 ± 7.2
			BMI (kg/m^2^)	NA	NA
			*→ two highest quintiles*		
Biolo et al. (2015)	BIA (Human IM-Plus, DS, Dieto System, BIA 101, Akern Srl, Tanita BC418MA, Tanita Corp.)	FM/FFM ratio > 0.8	*n* = 200	study performed in Italy and Slovenia	
				men	women
			*n*	89	111
			Age (year)	48.0 ± 12.0	51.0 ± 12.0
			BMI (kg/m^2^)	35.6 ± 6.2	35.5 ± 5.4
		
De Rosa et al. (2015)	BIA (Human IM Plus II–DS Medical)	SMI moderate and severe sarcopenia men: 8.44–9.53 kg/m^2^ and ≤8.43 kg/m^2^ women: 6.49–7.32 kg/m^2^ and ≤6.48 kg/m^2^ SMI (as % of body weight) moderate and severe sarcopenia men: 28.8–35.6% and ≤28.7% women: 23.1–28.4% and ≤23.0% SM by Janssen et al. (2000) equation + BMI ≥ 30 kg/m^2^	*n* = 500	Italians	
				men	women
			*n*	100	400
			Age (year)	27.0 ± 7.0	25.0 ± 6.0
			BMI (kg/m^2^)	25.8 ± 5.7	25.2 ± 5.7
			*→ moderate sarcopenia: within 1 to 2 SDs below the sex-specific means of young, healthy adults* *→ severe sarcopenia: 2 SDs below the sex-specific means of young, healthy adults*		
Atkins et al. (2014)	BIA (Bodystat 500, Bodystat Ltd.)	FFMI men: ≤16.7 kg/m^2^ FFM (equation by Deurenberg et al., 1991) + FMI > 11.1 kg/m^2^	*n* = 4045	study performed in UK (> 99 % white Europeans)	
				men	women
			*n*	4045	0
			Age (year)	60 to 79	
			BMI (kg/m^2^)	NA	
			*→ lowest two-fifths of FFMI*		
Baek et al. (2013)	BIA (InBody 520, Biospace)	ASMI men: 10.70 kg/m^2^ women: 8.60 kg/m^2^ + BMI > 25 kg/m^2^ (WHO definition)	*n* = 1150	study performed in Korea	
				men	women
			*n*	618	532
			Age (year)	43.6 ± 11.5	43.6 ± 11.5
			BMI (kg/m^2^)	24.6 ± 3.3	24.6 ± 3.3
			*→ 50th percentile of healthy study sample*		
Gomez-Cabello et al. (2011)	BIA (Tanita BC 418-MA)	(a) SMI men: 8.61 kg/m^2^ women: 6.19 kg/m^2^ (b) FM men: 30.33% women: 40.9% SM by Janssen et al. (2000) equation	*n* = 3136	Spaniards	
				men	women
			*n*	678	2198
			Age (year)	72.4 ± 5.5	72.1 ± 5.2
			BMI (kg/m^2^)	NA	NA
			*→ (a) two lower quintiles* *→ (b) two highest quintiles*		
Lou et al. (2017)	CT images	CT L3 SMI (Zhuang et al., 2016) men: ≤40.8 cm^2^/m^2^ women: ≤34.9 cm^2^/m^2^ + BMI ≥ 23 kg/m^2^ (WHO definition for Asians)	Predefined cut-off values for sarcopenia and obesity		
		
		
		
Ramachandran et al. (2012)	CT images (Somatom Sensation 10 CT scanner)	adjusted thigh muscle area: men: 110.7 cm^2^ women: 93.8 cm^2^ + (1) BMI ≥ 27 kg/m^2^ (2) WC ≥ 102 cm for men WC ≥ 88 cm for women	*n* = 539	study performed in US	
				men	women
			*n*	280	259
			Age (year)	71.1 ± 0.4	71.1 ± 0.4
			BMI (kg/m^2^)	NA	NA
			*→ lowest sex-specific tertile*		
Lim et al. (2010)	CT images (Brilliance 64, Philips)	Visceral fat area (VFA)/thigh muscle area (TMA) men: 0.93 women: 0.90	*n* = 264	Koreans	
				men	women
			*n*	126	138
			Age (year)	20 to 88	20 to 88
			BMI (kg/m^2^)	NA	NA
			*→ VFA/TMA median higher 50th percentile of the healthy study sample*		

ASM, appendicular skeletal muscle mass; ASMI, appendicular skeletal muscle mass index; BMI, body mass index; BIA, bioelectrical impedance analysis; CART, classification and regression tree analysis; CT, computed tomography; DXA, dual X-ray absorptiometry; FFM, fat-free mass; FFMI, fat-free mass index; FM, fat mass; FMI, fat mass index; FNIH, Foundation for the National Institutes of Health; IOTF, International Obesity Taskforce; L3, third lumbar vertebra; NA, not available; NIH, National Institutes of Health; SD, standard deviation; SM, skeletal muscle mass; SMI, skeletal muscle mass index; TAMA, total abdominal muscle area; TMA, thigh muscle area; VFA, visceral fat area; WC, waist circumference; WHO, World Health Organization.

**Table 5:** Generation of cut-offs for SMI (corresponding to BMI thresholds) based on FFMI.

	BMI (kg/m^2^)	FMI_DXA_ (kg/m^2^) (Kelly et al., 2009)	FFMI_DXA_ (kg/m^2^) (Modified according to Kelly et al., 2009)	SMI_MRI_ (kg/m^2^) (1.5 T Siemens Avanto MRI Scanner)	SMI_BIA_median_ (kg/m^2^) (mBCA 515, Seca)	SMI_BIA_-2SDs_ (kg/m^2^) (mBCA 515, Seca)
Caucasian men	<18.5	<2.9	15.6		8.6	>7.6
	>25	>6.0	19.0	9.85	9.7	>8.7
	>30	>8.9	21.1	10.71	10.5	>9.5
	>35	>11.9	23.1	12.15	11.4	>10.3
	>40	>15.0	25.0	13.67	12.2	>11.2
Caucasian women	<18.5	<4.9	13.6	6.65	6.7	>5.7
	>25	>9.2	15.8	7.49	7.7	>6.8
	>30	>12.9	17.1	8.15	8.5	>7.6
	>35	>16.8	18.2	8.99	9.3	>8.4
	>40	>20.6	19.4	9.74	10.1	>9.2

BMI, body mass index; FMI_DXA_, fat mass index by dual X-ray absorptiometry (QDR 4500A fan beam densitometer (Hologic, Inc., Bedford, MA, Hologic Discovery software version 12.1)); FFMI_DXA_, fat-free mass index by dual X-ray absorptiometry; SMI_MRI_, skeletal muscle mass index by magnetic resonance imaging calculated by stepwise regression analysis (*n* = 410, 219 women (age: 38 ± 13 years, BMI: 27.7 ± 6.5 kg/m^2^) and 191 men (age: 41 ± 14 years, BMI: 27.7 ± 5.0 kg/m^2^) (detailed description of the segmentation procedure given elsewhere (Schautz et al., 2012)); SMI_BIA_median_, skeletal muscle mass index by bioelectrical impedance analysis given as median calculated by linear regression analysis (*n* = 529, 264 women (27 ± 6 years, BMI: 23.9 ± 3.6 kg/m^2^) and 265 men (28 ± 6 years, BMI: 25.2 ± 3.2 kg/m^2^) (detailed description of the BIA measurement procedure given elsewhere (Bosy-Westphal et al., 2017)); SMI_BIA_-2SDs_, skeletal muscle mass index by bioelectrical impedance analysis given as 2 SDs below the sex-specific mean calculated as linear regression analysis.

## Abbreviation

ALM	appendicular lean mass
ASM	appendicular skeletal muscle mass
ASMI	appendicular skeletal muscle mass index
BIA	bioelectrical impedance analysis
BMI	body mass index
BSA	body surface area
CART	classification and regression tree analysis
CT	computed tomography
DXA	dual X-ray absorptiometry
FFM	fat-free mass
FFMI	fat-free mass index
FM	fat mass
FMI	fat mass index
FNIH	Foundation for the National Institutes of Health
IOTF	International Obesity Taskforce
L	lumbar vertebra
L3	third lumbar vertebra
MRI	magnetic resonance imaging
NA	not available
NAKO	German National Cohort
NHANES	National Health and Nutrition Examination Survey
NIH	National Institutes of Health
PMA	psoas muscle area
PMI	psoas muscle index
SAT	subcutaneous adipose tissue
SD	standard deviation
SEE	standard error of estimate
SM	skeletal muscle mass
SMI	skeletal muscle mass index
SMA	skeletal muscle area
T	thoracic vertebra
TAMA	total abdominal muscle area
TMA	thigh muscle area
VAT	visceral adipose tissue
VFA	visceral fat area
WC	waist circumference
WHO	World Health Organization
